# Real-World Serologic Responses and Recorded Medically Attended Events After the Live Attenuated MAV/06-Strain Varicella Vaccine in Adolescents and Adults: A Retrospective Cohort Study

**DOI:** 10.3390/vaccines14100875

**Published:** 2026-10-02

**Authors:** Yoon Kyung Cho, Yeji Kim, Gwanglok Kim, Raeseok Lee, Hyun Mi Kang

**Affiliations:** 1 Vaccine Bio Research Institute, College of Medicine, The Catholic University of Korea, Seoul 06591, Republic of Korea; 2 Department of Pediatrics, Seoul St. Mary’s Hospital, College of Medicine, The Catholic University of Korea, Seoul 06591, Republic of Korea; 3 Regulatory Science & Product Development Division, GC Biopharma Corporation, Yongin 16950, Republic of Korea; 4 Division of Infectious Diseases, Department of Internal Medicine, Seoul St. Mary’s Hospital, College of Medicine, The Catholic University of Korea, Seoul 06591, Republic of Korea

**Keywords:** varicella, varicella–zoster virus, live attenuated vaccine, MAV/06, adolescents, adults, immunogenicity, vaccine safety, real-world evidence

## Abstract

Background: Evidence on the real-world use of the MAV/06-strain live attenuated varicella vaccine (BARYCELA inj.) in adolescents and adults is limited. We described postvaccination ELISA seropositivity and recorded medically attended events in recipients aged ≥ 13 years. Methods: This retrospective cohort study included recipients vaccinated at a tertiary academic medical center in South Korea. Serologic analyses included selected participants documented as VZV IgG-negative at baseline who subsequently underwent postvaccination testing during routine care; dose scheduling and testing were individualized according to prior vaccination documentation and clinical need. Hospital encounters within 42 days after vaccination and institution-associated reports in the national adverse-event reporting system were reviewed. Results: Of 1715 administered doses, 988 patients aged ≥ 13 years received 1295 doses and comprised the safety cohort. The serology cohort included 199 participants and 202 dose-specific assessments. ELISA seropositivity was observed in 80/98 participants (81.6%; 95% CI, 72.8–88.1%) in the post-dose 1 cohort and 90/104 (86.5%; 95% CI, 78.7–91.8%) in the post-dose 2 cohort. Nine medically attended events were identified within 42 days (0.69% per administered dose; 95% CI, 0.32–1.32%), of which one mild fever episode was considered possibly related to vaccination. Substantial postvaccination ELISA seropositivity was observed in this selected cohort of adolescents and adults. Few medically attended events were identified in the available institutional records and national adverse-event reports, and one mild fever episode was considered possibly related to vaccination. Prospective studies with paired, protocol-timed sampling and cellular immune assessments are needed.

## 1. Introduction

Varicella is a highly contagious vaccine-preventable disease that can result in substantial morbidity among susceptible adolescents and adults, including severe skin infections, pneumonia, and neurologic complications [1,2,3]. Although universal childhood varicella vaccination has markedly reduced disease burden in countries with high vaccine coverage [4,5], immunity gaps persist in older age groups due to missed childhood vaccination, waning immunity, or the need for documented seroprotection before occupational exposure or initiation of immunosuppressive therapy [3,6,7]. In South Korea, BARYCELA inj. (GC Biopharma) is a live attenuated MAV/06-strain varicella vaccine licensed only for children under 13 years of age. While several varicella vaccines are formally licensed in Korea, they are not currently marketed or widely distributed for adolescents or adults. As a result, off-label use of BARYCELA inj. is increasingly common in clinical practice [8,9].

Safety data in older age groups are similarly sparse. Although large pediatric post-marketing studies of live attenuated varicella vaccines have characterized medically attended and reported events [10,11,12], the pattern of such events after the MAV/06-strain varicella vaccine in adolescents and adults, including those with underlying conditions, remains incompletely described [13,14,15]. Given the increasing clinical need for documented varicella immunity among healthcare workers, students, and patients recovering from immunosuppression, real-world data on serologic findings and recorded clinical events in these populations are warranted.

The present study addresses these knowledge gaps by describing vaccination patterns, postvaccination ELISA findings, and recorded medically attended events after the MAV/06-strain varicella vaccine in diverse clinical and occupational groups aged ≥ 13 years at a tertiary medical center. This study was not designed to characterize post-HSCT or post-chemotherapy recipients as separate clinical cohorts or to evaluate treatment-to-vaccination timing within those subgroups. Using serologic testing results, national adverse-event reports available to the institution, and electronic medical record review, this study provides real-world evidence in individuals aged ≥13 years. Because postvaccination testing occurred selectively during routine care and the dose-specific cohorts were largely distinct, the analyses were intended to be descriptive rather than to estimate a within-person dose effect.

## 2. Materials and Methods

### 2.1. Study Design and Setting

We conducted a retrospective cohort study of adolescents and adults who were prescribed BARYCELA injection (MAV/06 strain; GC Biopharma), a live attenuated varicella vaccine, under code DV-VZJ0.5 between May 2020 and July 2025. Postvaccination serologic data were collected through September 2025. Although BARYCELA inj. is licensed only for children <13 years of age, it is frequently used off-label in older individuals requiring documented varicella immunity. The Seoul St. Mary’s Hospital Institutional Review Board approved this study’s protocol (IRB No. KC25WASI0327, approval date: 23 May 2025) and waived the requirement for informed consent due to the retrospective nature of data collection.

### 2.2. Eligibility Criteria

Individuals were eligible for inclusion if they were ≥13 years of age at the time of vaccination, were prescribed DV-VZJ0.5 code at the study institution, and had available demographic and clinical information in the electronic medical record (EMR). For serologic analyses, additional criteria were a documented VZV IgG-negative result before vaccination and at least one VZV IgG result assigned to the post-dose 1 or post-dose 2 assessment. Participants were excluded if identifiers were incomplete, serologic testing was invalid, or vaccination was administered outside the study institution.

### 2.3. Data Collection

Demographic information, vaccination dates, clinical indications for vaccination, and medical history were reviewed from the institutional electronic medical record system. Serologic results, including prevaccination and postvaccination varicella–zoster virus (VZV) IgG results and specimen-collection dates, were retrieved from the hospital laboratory database. For the event analysis, institutional clinical encounters occurring within 42 days after vaccination were retrieved. Institution-associated reports in the national adverse-event reporting system were additionally screened for recipients aged < 18 years; adults were assessed through institutional encounter records. Consequently, events occurring outside the institution without an institution-associated report could not be ascertained. Under the institutional live-vaccine protocol, HSCT recipients were eligible for vaccination only when at least 24 months had elapsed since transplantation, the absolute CD4+ T-cell count was >200 cells/mm^3^, and no concurrent immunosuppressive therapy was being administered. Post-chemotherapy recipients were eligible only when at least 12 months had elapsed since completion of chemotherapy and they were not receiving immunosuppressive therapy.

### 2.4. Cohort Definitions

Two analytic datasets were constructed: (1) a safety cohort comprising 988 unique patients aged ≥ 13 years who received 1295 vaccine doses, analyzed descriptively per administered dose; and (2) a serology cohort comprising 199 participants with documented baseline VZV IgG-negative and postvaccination results, contributing 202 dose-specific assessments. In South Korea, the National Immunization Program includes one routine childhood varicella dose [9]. Consequently, vaccination pathways in older recipients were individualized. In otherwise healthy persons with a clearly documented childhood dose, one additional dose was often administered. When prior vaccination could not be verified and documented immunity was clinically or occupationally required—such as for healthcare personnel or clinically eligible persons with, or household contacts of persons with, immunocompromising conditions—clinicians could use a two-dose schedule consistent with risk-based guidance [3,6,7]. Postvaccination serology was ordered selectively for documentation or clinical decision-making rather than routinely after every dose. This practice contributed to the rarity of paired dose-specific serology. The post-dose 1 and post-dose 2 cohorts overlapped by only three participants and were therefore summarized as largely distinct dose-specific cohorts. No within-person incremental effect of dose 2 was estimated.

### 2.5. VZV IgG Assay and Serologic Assessment

Baseline VZV IgG-negative results from outside laboratories were accepted when documented in the medical record. Of the 199 participants, 97 (48.7%) had a baseline result marked as originating from an outside laboratory; assay manufacturer and cutoff information for these outside results were not available. All postvaccination VZV IgG results were measured using the VARICELLA–ZOSTER ELISA (Vircell, Granada, Spain). According to the manufacturer’s criteria, values > 11 IU/mL were positive, 9–11 IU/mL were equivocal, and <9 IU/mL were negative. The primary descriptive outcome was postvaccination ELISA seropositivity among participants documented as VZV IgG-negative before vaccination. Equivocal postvaccination results were classified as nonresponse. Dose-specific assessment intervals were defined in days between vaccination and the assigned serologic assessment, and summarized separately.

### 2.6. Ascertainment of Medically Attended Events

Medically attended events were identified through two mechanisms: (1) review of institutional clinical encounters within 42 days after vaccination, excluding routine visits for underlying disease or another vaccination, and (2) screening of institution-associated reports in the national adverse-event reporting system for recipients aged < 18 years. Events of interest included local reactions (injection-site pain, erythema, swelling, or rash), systemic events (fever, malaise, pneumonia, or hepatitis), immune-mediated conditions, neurologic syndromes, herpes zoster, hospitalization, and death. Each identified event was clinically reviewed and categorized as related, possibly related, or unrelated to vaccination. This strategy was designed to identify documented medically attended or reported events and was not capable of measuring the overall frequency of mild local or systemic reactions that did not lead to an encounter or report.

### 2.7. Statistical Analysis

Continuous variables were summarized as median (interquartile range [IQR]) and categorical variables as frequency (percentage). Two-sided Wilson 95% confidence intervals were calculated for postvaccination ELISA-seropositivity proportions. Exact two-sided Clopper–Pearson 95% confidence intervals were calculated for recorded medically attended-event proportions. No inferential comparison was made between the post-dose 1 and post-dose 2 proportions because the cohorts were largely distinct and the clinically relevant within-person dose effect could not be estimated. Age was compared using the two-sided Mann–Whitney U test, binary variables using the two-sided Fisher exact test, and the three-category vaccination-indication variable using the two-sided Fisher–Freeman–Halton exact test; these analyses were exploratory. Predictors of nonresponse were evaluated separately in each dose-specific cohort using multivariable Firth penalized logistic regression. Odds-ratio confidence intervals and *p*-values were based on the penalized profile likelihood. All *p*-values were two-sided and nominal. Analyses were reproduced using Python 3.11.15.

## 3. Results

### 3.1. Study Participants

**Figure 1:**
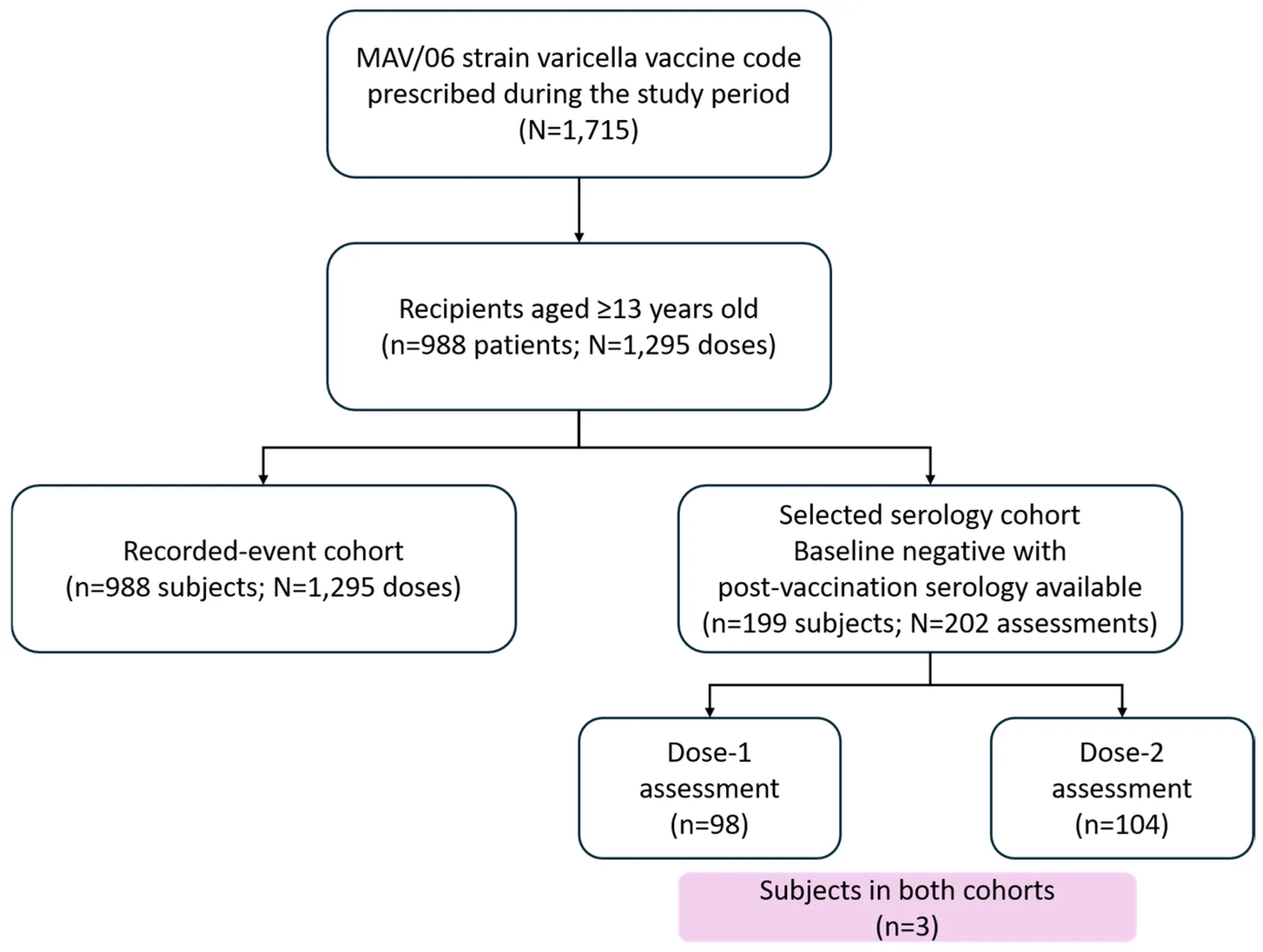
Study cohorts. Among 1715 doses prescribed during the study period, 988 unique patients aged ≥ 13 years received 1295 doses and comprised the safety cohort. The selected serology cohort included 199 participants (20.1% of the safety cohort), contributing 202 assessments: dose 1, *n* = 98; dose 2, *n* = 104; both cohorts, *n* = 3. Postvaccination serology was obtained selectively during clinically driven follow-up. Dose-specific estimates are not paired within-person responses.

During the study period, 1715 doses of the vaccine were prescribed. The safety cohort comprised 988 unique patients aged ≥ 13 years who received a total of 1295 doses. Safety outcomes were summarized per administered dose. Of the 988 patients, 199 (20.1%) had a documented baseline VZV IgG-negative result and at least one postvaccination serologic result, contributing 202 dose-specific assessments to the selected serology cohort (Figure 1). Post-dose 1 and post-dose 2 results were available for 98 and 104 participants, respectively, with only three participants represented in both cohorts. The median age was 20 years (IQR, 17–24 years); 58 participants (29.1%) were aged 13–17 years, 109 (54.8%) were aged 18–29 years, and only five (2.5%) were aged ≥ 40 years. Overall, 83 participants (41.7%) were male, and 101 (50.8%) had an underlying medical condition. Vaccination-related clinical categories were post-HSCT (*n* = 57, 28.6%), hospital employees undergoing serologic screening (*n* = 46, 23.1%), post-chemotherapy (*n* = 41, 20.6%), other serologic screening (*n* = 32, 16.1%), and medical or nursing students (*n* = 23, 11.6%) (Table 1). Among 101 participants with both dose dates recorded, the median dose 1-to-dose 2 interval was 65 days (IQR, 35–125; range, 28–406 days).

### 3.2. Postvaccination ELISA Seropositivity

**Figure 2:**
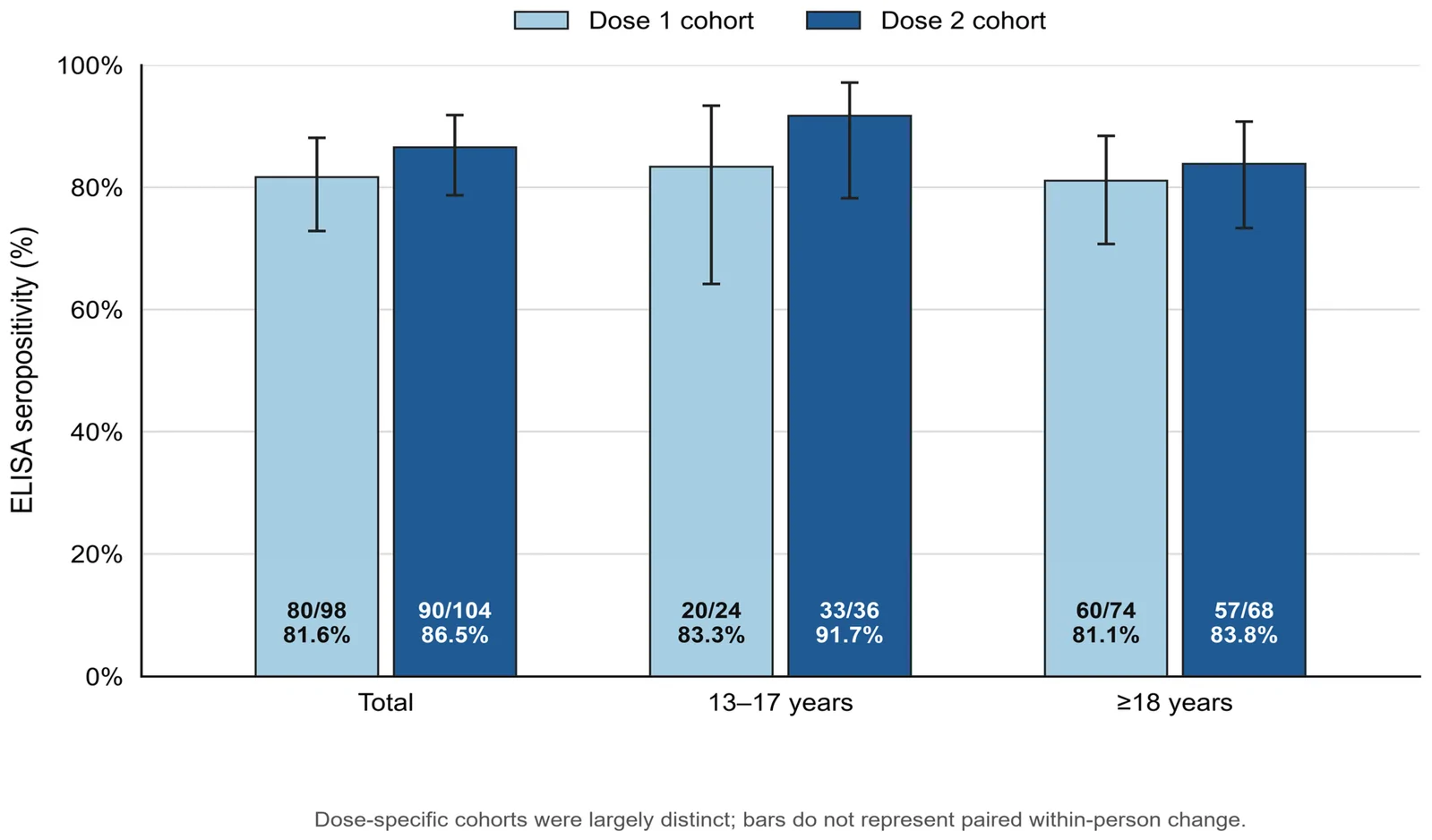
Postvaccination ELISA seropositivity in the dose 1 and dose 2 cohorts, both overall and by age group. Bars show *n*/N (%) and vertical error bars show the 95% confidence intervals. Equivocal results were classified as nonresponse.

Figure 2 shows postvaccination ELISA seropositivity in the post-dose 1 and post-dose 2 cohorts, both overall and by age group. A positive result was observed in 80 of 98 participants in the post-dose 1 cohort (81.6%; 95% CI, 72.8–88.1%) and 90 of 104 participants in the post-dose 2 cohort (86.5%; 95% CI, 78.7–91.8%). These largely unpaired proportions describe two selected dose-specific cohorts and do not estimate the incremental effect of a second dose within the same individuals. The median assessment interval was 188 days after dose 1 (IQR, 63–519; range, 26–1350 days) and 146 days after dose 2 (IQR, 63–476; range, 22–1559 days). The wide ranges reflect non-protocolized testing during routine care. In exploratory adjusted logistic models, assessment interval was not clearly associated with ELISA seropositivity in either cohort (per 180 additional days: dose 1 OR, 0.98; 95% CI, 0.71–1.34; *p* = 0.888; dose 2 OR, 1.01; 95% CI, 0.77–1.31; *p* = 0.960). In sensitivity analyses of equivocal-result handling, excluding equivocal results yielded ELISA-seropositivity proportions of 87.9% (80/91; 95% CI, 79.6–93.1%) for the post-dose 1 cohort and 89.1% (90/101; 95% CI, 81.5–93.8%) for the post-dose 2 cohort. Classifying equivocal results as positive yielded 88.8% (87/98; 95% CI, 81.0–93.6%) and 89.4% (93/104; 95% CI, 82.0–94.0%), respectively.

**Figure 3:**
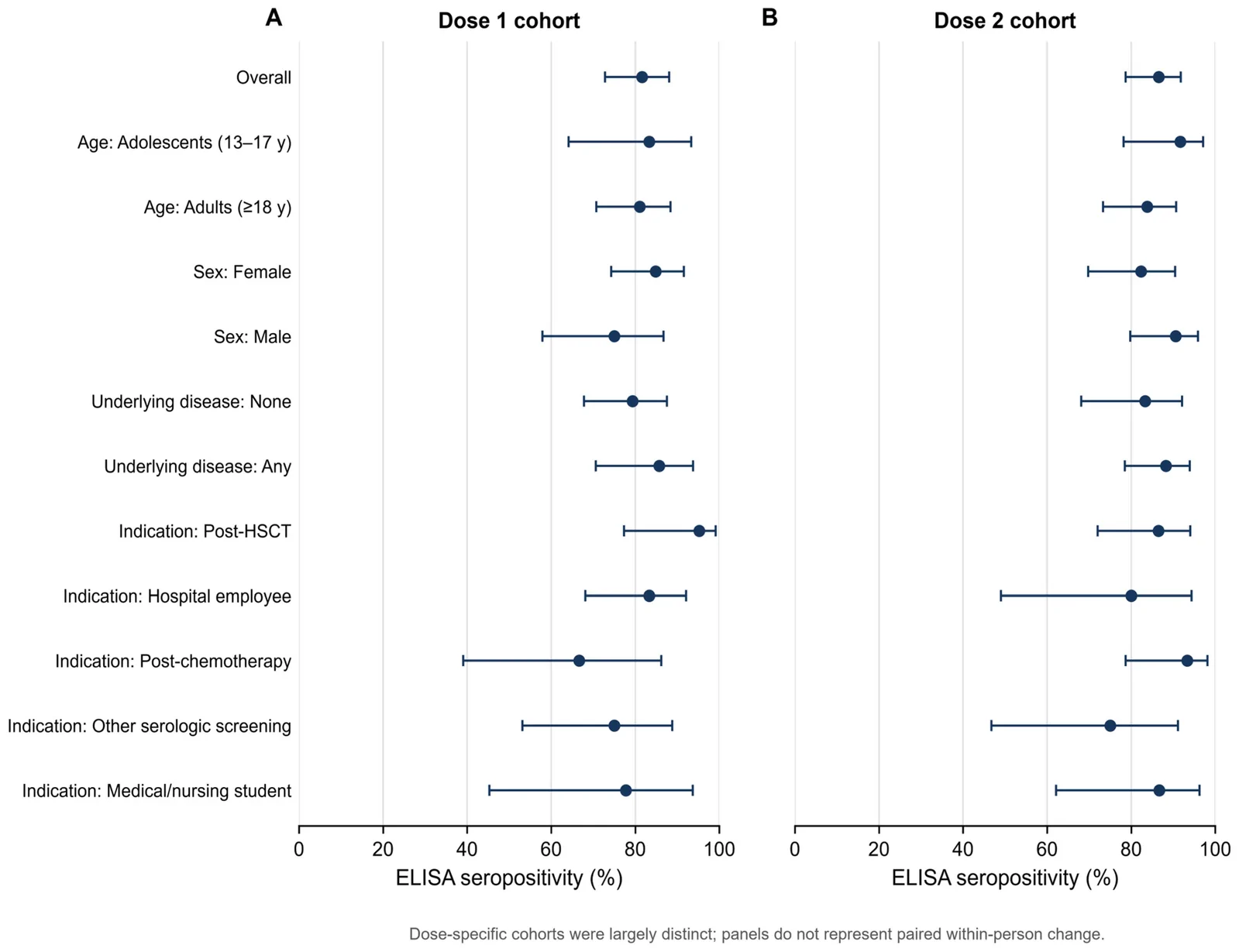
Postvaccination ELISA seropositivity in the (**A**) post-dose 1 and (**B**) post-dose 2 cohorts according to demographic and clinical subgroups. Open circles indicate point estimates, and horizontal error bars indicate Wilson 95% confidence intervals. A positive postvaccination VZV IgG result was assessed among participants documented as negative at baseline; equivocal postvaccination results were classified as nonresponse. HSCT, hematopoietic stem-cell transplantation;.

Subgroup-specific postvaccination ELISA-seropositivity proportions are presented in Figure 3. In the post-dose 1 cohort, a positive result was observed in 83.3% of adolescents (20/24; 95% CI, 64.1–93.3%) and 81.1% of adults (60/74; 95% CI, 70.7–88.4%). In the post-dose 2 cohort, the corresponding proportions were 91.7% among adolescents (33/36; 95% CI, 78.2–97.1%) and 83.8% among adults (57/68; 95% CI, 73.3–90.7%). Across clinical-indication subgroups, estimates ranged from 66.7% among post-chemotherapy participants to 95.2% among post-HSCT participants in the post-dose 1 cohort, and from 75.0% among participants undergoing other serologic screening to 93.3% among post-chemotherapy participants in the post-dose 2 cohort. Several subgroups were small, and confidence intervals were wide; these findings are descriptive and do not establish differences between subgroups or between doses.

In the post-dose 1 cohort (*n* = 98; 18 nonresponses), Firth penalized logistic regression did not identify clear associations between nonresponse and age (odds ratio [OR] per 1-year increase, 1.02; 95% confidence interval [CI], 0.94–1.10; *p* = 0.562), male sex (OR, 1.91; 95% CI, 0.68–5.35; *p* = 0.217), or post-HSCT or post-chemotherapy indication versus other indications (OR, 0.93; 95% CI, 0.21–3.81; *p* = 0.921). In the post-dose 2 cohort (*n* = 104; 14 nonresponses), the corresponding ORs were 1.00 per 1-year increase in age (95% CI, 0.82–1.17; *p* = 0.958), 0.59 for male versus female sex (95% CI, 0.17–1.86; *p* = 0.368), and 0.56 for post-HSCT or post-chemotherapy indication versus other indications (95% CI, 0.12–2.35; *p* = 0.433). Given the sparse events and wide confidence intervals, these exploratory models do not establish the absence of associations (Table 2 and Table 3).

### 3.3. Recorded Medically Attended Events

Among 1295 administered doses in 988 patients, nine medically attended events were identified in institutional records or institution-associated reports within 42 days after vaccination (0.69% per dose; 95% confidence interval [CI], 0.32–1.32%) (Table 4). Two fever-associated encounters were recorded (0.15%; 95% CI, 0.02–0.56%), including one mild fever episode on day 4 (0.08%; 95% CI, 0.002–0.43%). No life-threatening, hospitalization-requiring, herpes zoster, immune-mediated, neurologic, or fatal events were recorded; for each zero-event outcome, the upper limit of the exact 95% CI was 0.28%. The nine events occurred between days 4 and 41 and included mild fever, sinusitis, hypothyroidism, acute gastroenteritis, viral upper respiratory infection, fever with injection-site pain after subsequent COVID-19 vaccination, blepharitis, urinary tract infection, and aggravation of atopic dermatitis (Table 5). Based on clinical review, the mild fever episode on day 4 was considered possibly related to vaccination, whereas the remaining events were considered unrelated.

## 4. Discussion

This retrospective cohort provides, to our knowledge, one of the first real-world descriptions of postvaccination ELISA seropositivity and medically attended safety events following the MAV/06-strain live attenuated varicella vaccine (BARYCELA inj.) used off-label in adolescents and adults in South Korea, a population and use-case with limited existing data. A positive postvaccination ELISA result was observed in 81.6% of the post-dose 1 cohort and 86.5% of the post-dose 2 cohort, results that are consistent with expectations from the existing Oka-strain literature and support the immunogenicity of MAV/06 in this setting.

The post-dose 1 seropositivity proportion (81.6%) falls within the range historically reported after a single Oka-strain varicella-vaccine dose in adults [16], reinforcing the biological plausibility of these findings. The post-dose 2 cohort showed a numerically higher proportion (86.5%); while the largely distinct composition of the two cohorts, reflecting routine clinical practice rather than a planned longitudinal schedule, means that this difference cannot be interpreted as within-person boosting, the finding is directionally consistent with expected vaccine biology and provides a reasonable basis for prospective confirmation.

As with any serology-based real-world study, a negative ELISA result does not fully exclude prior immunity: commercial ELISAs can be less sensitive than FAMA for detecting vaccine-induced VZV antibody, and antibody alone may not capture the full picture of VZV-specific immune memory [6,15,17,18]. Ninety-seven participants entered the cohort with baseline negativity documented by an outside laboratory; and although the manufacturer and cutoff details for these external results were not available, this reflects the pragmatic, multi-site nature of real-world data collection.

One biologically plausible explanation for these proportions is that vaccination acted as antigenic re-exposure in individuals carrying pre-existing VZV memory that baseline ELISA did not detect [19,20]. While our dataset, which lacks T-cell response data, antibody avidity, and complete prior vaccination history, cannot confirm this mechanism, our findings are consistent with it and provide a valuable empirical foundation for prospective mechanistic studies.

The age composition of the cohort follows from Korean seroepidemiology rather than from selective recruitment. A nationwide seroepidemiologic study of varicella–zoster virus in Korea reported VZV IgG seropositivity of 71% among 15–19-year-olds and 76% among 20–24-year-olds, rising above 95% after age 40 [21]. The susceptible population that screening identifies and that clinicians then vaccinate is concentrated in adolescence and early adulthood, which is where our participants fall (median age of 20 years; five participants aged ≥ 40 years and none aged ≥ 60). Our findings therefore apply most directly to adolescents and young adults; immunogenicity and safety in middle-aged and older adults remain to be established, and we identify that group as a priority for future study.

Only nine medically attended events were identified within 42 days among 1295 safety records, and no life-threatening, hospitalization-requiring, neurologic, immune-mediated, herpes zoster, or fatal events were recorded. One mild fever episode was considered possibly related to vaccination. This pattern is broadly consistent with post-licensure experience with Oka-strain live attenuated varicella vaccines [10,11,12,22,23,24]. However, these findings should be interpreted as the frequency of recorded medically attended events rather than as a complete estimate of reactogenicity or vaccine-attributable risk, because mild events not resulting in clinical encounters may have been missed and the safety dataset was record-level.

Several limitations should be considered when interpreting these findings. This study reflects individualized, clinically driven vaccination and follow-up rather than a prospective research protocol or a licensure trial; this captured authentic real-world practice, but it also determined who entered the serology cohort. Second, the dose-specific cohorts overlapped by only three participants and were therefore largely distinct, which precludes any within-individual assessment of boosting. Furthermore, due to the retrospective nature of this study, serologic assessment was also nonuniform: baseline negativity was documented by outside laboratories in roughly half the cohort with the assay and cutoff unrecorded; the commercial ELISA may underestimate detectable VZV antibody relative to FAMA; and no FAMA, quantitative antibody, avidity, or cell-mediated immunity measurements were available. Another limitation was that assessment timing varied widely, and although exploratory adjusted models did not identify a clear association between the vaccination-to-serology interval and seropositivity, cross-sectional measurements of this kind cannot exclude antibody waning. On the safety side, the retrospective, national adverse-event reports were screened only for recipients aged <18 years, whereas event ascertainment in adults relied on institutional encounter records; capture, therefore, differed by age, and events occurring outside the institution without documentation in the institutional record were not ascertainable in adults. Also, transplantation and chemotherapy-completion dates were not abstracted as analytic variables, so treatment-to-vaccination intervals could not be characterized in the post-HSCT and post-chemotherapy subgroups. Finally, clinical protection against varicella was not assessed.

To conclude, in this cohort of adolescents and adults documented as VZV IgG-negative before vaccination, postvaccination ELISA-seropositivity was observed in 81.6% of the post-dose 1 cohort and 86.5% of the largely distinct post-dose 2 cohort. Only a few medically attended events were identified in the available institutional records and reports, of which one mild fever episode was considered possibly related to vaccination. Taken together, this study offers meaningful, real-world evidence supporting the immunogenicity and preliminary safety profile of MAV/06 in adolescents and adults, and provides a strong empirical basis for prospective evaluation. Future studies with a defined vaccinated population; paired baseline and post-dose specimens at protocol-specified times; sensitive humoral and cellular assays; and active solicitation of clinical events will help confirm and extend these findings. The present estimates should not be used to infer within-person two-dose benefit or to define the full safety profile beyond the scope of recorded-event ascertainment, but they represent a valuable and timely contribution given the current scarcity of real-world MAV/06 data.

## Figures and Tables

**Table 1:** Baseline characteristics of participants included in the postvaccination ELISA-seropositivity analyses.

Characteristic	Total
	N = 199
Age, years	
Median (IQR)	20 (17–24)
Age distribution, *n* (%)	
13–17 years	58 (29.1%)
18–29 years	109 (54.8%)
30–39 years	27 (13.6%)
40–49 years	3 (1.5%)
50–59 years	2 (1.0%)
≥60 years	0
Male, *n* (%)	83 (41.7%)
Underlying medical condition, *n* (%)	101 (50.8%)
Reason for screening, *n* (%)	
Post-hematopoietic stem-cell transplantation	57 (28.6%)
Hospital employee	46 (23.1%)
Post-chemotherapy	41 (20.6%)
Identified during serologic screening for other purposes	32 (16.1%)
Medical or nursing student	23 (11.6%)

Abbreviations: IQR, interquartile range.

**Table 2:** Exploratory predictors of nonresponse in the post-dose 1 and post-dose 2 cohorts.

Dose	N	Events	Predictor	OR	95% CI	PLR *p*
1	98	18	Age, per 1-year increase	1.02	0.94–1.10	0.562
			Male sex (vs. female)	1.91	0.68–5.35	0.217
			Post-HSCT or post-chemotherapy indication (vs. other indications)	0.93	0.21–3.81	0.921
2	104	14	Age, per 1-y increase	1.00	0.82–1.17	0.958
			Male sex (vs. female)	0.59	0.17–1.86	0.368
			Post-HSCT or post-chemotherapy indication (vs. other indications)	0.56	0.12–2.35	0.433

Abbreviations: CI, confidence interval; HSCT, hematopoietic stem-cell transplantation; OR, odds ratio; PLR, penalized profile-likelihood ratio. Nonresponse included negative and equivocal results. Confidence intervals and *p*-values were obtained from the penalized profile likelihood.

**Table 3:** Characteristics of participants by serologic response status after dose 1 and dose 2 of BARYCELA.

Characteristic	Dose 1 Responders (*n* = 80)	Dose 1 Nonresponders (*n* = 18)	*p* Value	Dose 2 Responders (*n* = 90)	Dose 2 Nonresponders (*n* = 14)	*p*-Value
Age, median (IQR), y	23 (18–30)	27.5 (19–32)	0.295	19 (15–21)	19 (18–20)	0.569
Male sex, no. (%)	24 (30.0)	8 (44.4)	0.272	48 (53.3)	5 (35.7)	0.260
Underlying disease, no. (%)	30 (37.5)	5 (27.8)	0.588	60 (66.7)	8 (57.1)	0.551
Post-HSCT indication, no. (%)	20 (25.0)	1 (5.6)	—	32 (35.6)	5 (35.7)	—
Post-chemotherapy indication, no. (%)	8 (10.0)	4 (22.2)	—	28 (31.1)	2 (14.3)	—
Other indications, no. (%)	52 (65.0)	13 (72.2)	0.097 *	30 (33.3)	7 (50.0)	0.320 *

Abbreviations: HSCT, hematopoietic stem-cell transplantation; IQR, interquartile range. Equivocal results were classified as nonresponse. Other indications comprised hospital employees, medical or nursing students, and participants undergoing serologic screening for other purposes. For age, *p*-values were calculated using the two-sided Mann–Whitney U test, and those for binary variables were calculated using the two-sided Fisher exact test. Asterisks (*) identify the two-sided Fisher–Freeman–Halton exact *p*-value for the global comparison across the three mutually exclusive indication categories (post-HSCT, post-chemotherapy, and other indications). Em dashes indicate that no category-specific *p*-value was calculated for the individual post-HSCT and post-chemotherapy rows.

**Table 4:** Recorded medically attended events within 42 days after vaccination.

Endpoint	Events	Administered Doses	Rate, %	95% Confidence Interval
Any medically attended event within 42 day	9	1295	0.695	0.318–1.315
Any fever-associated encounter within 42 day	2	1295	0.154	0.019–0.558
Mild fever encounter on day 4	1	1295	0.077	0.002–0.430
Event coded life-threatening	0	1295	0.000	0.000–0.284
Event coded as requiring hospitalization	0	1295	0.000	0.000–0.284
Herpes zoster event recorded	0	1295	0.000	0.000–0.284
Immune-mediated event recorded	0	1295	0.000	0.000–0.284
Neurologic event recorded	0	1295	0.000	0.000–0.284
Fatal event recorded	0	1295	0.000	0.000–0.284

Event-ascertainment yields and exact two-sided Clopper–Pearson 95% confidence intervals are shown per 1295 administered doses among 988 unique patients; these values do not estimate overall reactogenicity or vaccine-attributable risk.

**Table 5:** Timeline and assessed relatedness of recorded medically attended events.

Case No.	Sex	Age	Onset_Day	Vaccine Dose Preceding Event/Total Doses	Event	Vaccine Relatedness
1	F	34	4	1/1	Fever, mild (<38.3 °C)	Possibly related *
2	F	33	13	1/1	Sinusitis	Unrelated
3	M	30	16	1/1	Hypothyroidism	Unrelated
4	M	15	27	2/2	Acute gastroenteritis	Unrelated
5	M	23	28	1/1	Viral upper-respiratory infection	Unrelated
6	F	19	33	2/2	Fever and injection-site pain after COVID-19 vaccination (3 days prior)	Unrelated
7	F	22	36	2/2	Blepharitis	Unrelated
8	F	26	41	1/1	UTI	Unrelated
9	M	32	41	1/1	Atopic dermatitis aggravation	Unrelated

Abbreviation: COVID-19, coronavirus disease 2019. * The live attenuated varicella vaccine was administered without co-administration of other vaccines.

## Data Availability

The data are not publicly available, because they contain potentially identifiable clinical information and are subject to institutional privacy restrictions. De-identified data may be available from the corresponding author upon reasonable request and with institutional approval.

## Abbreviations

The following abbreviations are used in this manuscript:

AEFI	adverse event following immunization
CI	confidence interval
ELISA	enzyme-linked immunosorbent assay
FAMA	Fluorescent antibody to membrane antigen
HSCT	hematopoietic stem-cell transplantation
IQR	interquartile range
VZV	varicella–zoster virus.
